# Supplementary material for: Heat adaptation of phage T7 under an extended genetic code
Source: Virus Evol. 2021 Dec 1;7(2):veab100. doi: 10.1093/ve/veab100 (PMC8923235; doi:10.1093/ve/veab100)
Supplement: veab100_Supp [file veab100_supp.zip › Sequencing_Analysis.zip!/Sequencing_Analysis/S1/evidence/JC_46.html]

BRESEQ :: Evidence


| Predicted mutation | | | | | | |
| --- | --- | --- | --- | --- | --- | --- |
| evidence | seq id | position | mutation | annotation | gene | description |
| MC JC | NC\_001604.1 | 1,253 | Δ1,489 bp | intergenic (–/–) | – / – | –/– |

| Missing coverage evidence... | | | | | | | | | | |
| --- | --- | --- | --- | --- | --- | --- | --- | --- | --- | --- |
|  |  |  | seq id | start | end | size | ←reads | reads→ | gene | description |
| \* | \* | ÷ | NC\_001604.1 | 1253 | 2741 | 1489 | 4499 [53] | [87] 4530 | *–/–* | –/– |

| New junction evidence | | | | | | | | | | | |
| --- | --- | --- | --- | --- | --- | --- | --- | --- | --- | --- | --- |
|  | | seq id | position | reads (cov) | reads (cov) | score | skew | freq | annotation | gene | product |
| \* | ? | NC\_001604.1 | = 1252 | 53 (0.010) | 4384 (1.080) | 395/580 | 0.0 | 98.5% | intergenic (–/–) | *–/–* | –/– |
|  | ? | NC\_001604.1 | 2742 = | 84 (0.020) |  |  |  |  | intergenic (–/–) | *–/–* | –/– |

Only 100 of 4651 total aligned reads displayed.  

`ACTTACAACAACGTTTTCGACCACGCTTACGAAATGCTGAAAGAAAACATCCGTTATGATGACATCCGTGACACTGATGACCTGCACGATGCTATTCACATGGCTGCCGATAATGCAGTTCCGCACTACTACGCTGACATCTTTAGCGTAATGGCAAGTGAGGGCATTGACCTTGAGTTCGAAGACTCTGGTCTGATGCCTGACACCAAGGACGTAATCCGCATCCTGCAAGCGCGTATCTATGAGCAATTAACGATTGACCTCTGGGAAGACGCAGAAGACTTGCTCAATGAATACTT‑‑‑‑‑‑‑G‑‑‑‑‑‑‑‑‑GAGGAA‑‑‑‑‑‑‑‑‑GTCG‑‑‑‑‑‑‑‑‑‑‑‑‑‑‑‑‑‑‑‑‑‑‑‑‑‑‑‑‑‑‑‑‑‑‑‑‑‑‑‑‑‑‑‑‑‑‑‑‑‑‑‑‑‑‑‑‑‑‑‑‑‑‑‑‑‑‑‑‑‑‑‑‑‑‑‑‑‑‑‑‑‑‑‑‑‑‑‑‑‑‑‑‑‑‑‑‑‑‑‑‑‑‑‑‑‑‑‑‑‑‑‑‑‑‑‑‑‑‑‑‑‑‑‑‑‑‑‑‑‑‑‑‑‑‑‑‑‑‑‑‑‑‑‑‑‑‑‑‑‑‑‑‑‑‑‑‑‑‑‑‑‑‑‑‑‑‑‑‑‑‑‑‑‑‑‑‑‑‑‑‑‑‑‑‑‑‑‑‑‑‑‑‑‑‑‑‑‑‑‑‑‑‑‑‑‑‑‑‑‑‑‑‑‑‑‑‑‑‑‑‑‑‑‑‑‑‑‑‑‑‑‑‑‑‑‑‑‑‑‑‑‑‑‑‑‑‑‑‑‑‑‑‑‑‑‑‑‑‑‑‑‑‑‑‑‑‑‑‑‑‑‑‑‑‑‑‑‑‑‑‑‑‑‑‑‑‑‑‑‑‑‑‑‑‑‑‑‑‑‑‑‑‑‑‑‑‑  >  NC_001604.1/943‑1252`  
`‑‑‑‑‑‑‑‑‑‑‑‑‑‑‑‑‑‑‑‑‑‑‑‑‑‑‑‑‑‑‑‑‑‑‑‑‑‑‑‑‑‑‑‑‑‑‑‑‑‑‑‑‑‑‑‑‑‑‑‑‑‑‑‑‑‑‑‑‑‑‑‑‑‑‑‑‑‑‑‑‑‑‑‑‑‑‑‑‑‑‑‑‑‑‑‑‑‑‑‑‑‑‑‑‑‑‑‑‑‑‑‑‑‑‑‑‑‑‑‑‑‑‑‑‑‑‑‑‑‑‑‑‑‑‑‑‑‑‑‑‑‑‑‑‑‑‑‑‑‑‑‑‑‑‑‑‑‑‑‑‑‑‑‑‑‑‑‑‑‑‑‑‑‑‑‑‑‑‑‑‑‑‑‑‑‑‑‑‑‑‑‑‑‑‑‑‑‑‑‑‑‑‑‑‑‑‑‑‑‑‑‑‑‑‑‑‑‑‑‑‑‑‑‑‑‑‑‑‑‑‑‑‑‑‑‑‑‑‑‑‑‑‑‑‑‑‑‑‑‑‑‑‑‑‑‑‑‑‑‑‑‑‑‑‑‑‑‑‑‑‑‑‑‑‑‑‑‑‑‑‑‑‑‑‑‑‑‑‑‑‑‑‑‑‑‑‑‑‑‑‑‑‑‑‑‑‑‑‑‑‑‑‑‑‑‑gaggaa‑‑‑‑‑‑‑‑‑gtcg‑‑‑‑‑‑‑C‑‑ACGACAGAAAGAAATTGACCGCGCTAAGGCCCGTAAAGAACGTCACGAGGGGCGCTTAGAGGCACGCAGATTCAAACGTCGCAACCGCAAGGCACGTAAAGCACACAAAGCTAAGCGCGAAAGAATGCTTGCTGCGTGGCGATGGGCTGAACGTCAAGAACGGCGTAACCATGAGGTAGCTGTAGATGTACTAGGAAGAACCAATAACGCTATGCTCTGGGTCAACATGTTCTCTGGGGACTTTAAGGCGCTTGAGGAACGAATCGCGCTGCACTGGCGTAATGCTGACCGGATGGC  >  NC_001604.1/2742‑3039`  

                                                                                                                                                                                                                                                                                                                                                                                                                                                                                                                                                                                                                                                                    
 `CTTACAACAACGGTTTCGACCACGCTTACGAAATGCTGAAAGAAAACATCCGTTATGATGACATCCGTGACACTGATGACCTGCACGATGCTATTCACATGGCTGCCGATAATGCAGTTCCGCACTACTACGCTGACATCTTTAGCGTAATGGCAAGTGAGGGCATTGACCTTGAGTTCGAAGACTCTGGTCTGATGCCTGACACCAAGGACGTAATCCGCATCCTGCAAGCGCGTATCTATGAGCAATTAACGATTGACCTCTGGGAAGACGCAGAAGACTTGCTCAATGAATACTT‑‑‑‑‑‑‑G‑‑‑‑‑‑‑‑‑GA                                                                                                                                                                                                                                                                                                                                      <  1:264720/301‑1`  
   `TACAACAGCGTTTTCGACCACGCTTACGAAATGCTGAAAGAAAACATCCGTTATGATGACATCCGTGACACTGATGACCTGCACGATGCTATTCACATGGCTGCCGATAATGCAGTTCCGCACTACTACGCTGACATCTTTAGCGTAATGGCAAGTGAGGGCATTGACCTTGAGTTCGAAGACTCTGGTCTGATGCCTGACACCAAGGACGTAATCCGCATCCTGCAAGCGCGTATCTATGAGCAATTAACGATTGATCTCTGGGAAGACGCAGAAGACTTGCTCAATGAATACTT‑‑‑‑‑‑‑G‑‑‑‑‑‑‑‑‑GAGG                                                                                                                                                                                                                                                                                                                                    <  1:139493/301‑1`  
               `TTCGACCACGCTTACGAAATGCTGAAAGAAAACATCCGTTATGAGGACATCCGTGACACTGATGACCTGCACGATGCTATTCACATGGCTGCCGATAATGCAGTTCCGCACTACTACGCTGACATCTTTAGCGTAATGGCAAGTGAGGGCATTGACCTTGAGTTCGAAGACTCTGGTCTGATGCCTGACACCAAGGACGTAATCCGCATCCTGCAAGCGCGTATCTATGAGCAATTAACGATTGACCTCTGGGAAGACGCAGAAGACTTGCTCAATGAATACTT‑‑‑‑‑‑‑G‑‑‑‑‑‑‑‑‑GAGGAA‑‑‑‑‑‑‑‑‑GGCG‑‑‑‑‑‑‑C‑‑ACGAC                                                                                                                                                                                                                                                                                                      <  1:422922/301‑1`  
                      `ataagagacagATGCTGAAAGAAAACATCCGTTATGATGACATCCGTGACACTGATGACCTGCACGATGCTATTCACATGGCTGCCGATAATGCAGTTCCGCACTACTACGCTGACATCTTTAGCGTAATGGCAAGTGAGGGCATTGACCTTGAGTTCGAAGATTCTGGTCTGATGCCTGACACCAAGGACGTAATCCGCATCCTGCAAGCGCGTATCTATGAGCAATTAACGATTGACCTCTGGGAAGACGCAGAAGACTTGCTCAATGAATACTT‑‑‑‑‑‑‑G‑‑‑‑‑‑‑‑‑GAGGAA‑‑‑‑‑‑‑‑‑GTCG‑‑‑‑‑‑‑C‑‑ACGACAGAAAGA                                                                                                                                                                                                                                                                                               <  1:298724/290‑1`  
                       `CGCTTACGAAATGCTGAAAGAAAACATCCGTTATGATGACATCCGTGACACTGATGACCTGCACGATGCTATTCACATGGCTGCCGATAATGCAGTTCCGCACTACTACGCTGACATCTTTAGCGTAATGGCAAGTGAGGGCATTGACCTTGAGTTCGAAGACTCTGGTCTGATGCCTGACACCAAGGACGTAATCCGCATCCTGCAAGCGCGTATCTATGAGCAATTAACGATTGACCTCTGGGAAGACGCAGAAGACTTGCTCAATGAATACTT‑‑‑‑‑‑‑G‑‑‑‑‑‑‑‑‑GAGGAA‑‑‑‑‑‑‑‑‑GTCG‑‑‑‑‑‑‑C‑‑ACGACctgtctct                                                                                                                                                                                                                                                                                              >  1:210371/1‑293`  
                        `ggggctcggagatgggaataagagacagGTTATGATGACATCCGTGACACTGATGACCTGCACGATGCTATTCACATGGCTGCCGATAATGCAGTTCCGCACTACTACGCTGACATCTTTAGCGTAATGGCAAGTGAGGGCATTGACCTTGAGTTCGAAGACTCTGGTCTGATGCCTGACACCAAGGACGTAATCCGCATCCTGCAAGCGCGTATCTATGAGCAATTAACGATTGACCTCTGGGAAGACGCAGAAGACTTGCTCAATGAATACTT‑‑‑‑‑‑‑G‑‑‑‑‑‑‑‑‑GAGGAA‑‑‑‑‑‑‑‑‑GTCG‑‑‑‑‑‑‑C‑‑ACGACAGAAAGAAA                                                                                                                                                                                                                                                                                             <  1:329314/273‑1`  
                         `CTTACGAAATGCTGAAAGAAAACATCCGTTATGATGACATCCGTGACACTGATGACCTGCACGATGCTATTCACATGGCTGCCGATAATGCAGTTCCGCACTACTACGCTGACATCTTTAGCGTAATGGCAAGTGAGGGCATTGACCTTGAGTTCGAAGACTCTGGTCTGATGCCTGACACCAAGGACGTAATCCGCATCCTGCAAGCGCGTATCTATGAGCAATTAACGATTGACCTCTGGGAAGACGCAGAAGACTTGCTCAATGAATACTT‑‑‑‑‑‑‑G‑‑‑‑‑‑‑‑‑GAGGAA‑‑‑‑‑‑‑‑‑GTCG‑‑‑‑‑‑‑C‑‑ACGACAGAAAGAAAT                                                                                                                                                                                                                                                                                            >  1:379292/1‑301`  
                            `ACGAAATGCTGAAAGAAAACATCCGTTATGATGACATCCGTGACACTGATGACCTGCACGATGCTATTCACATGGCTGCCGATAATGCAGTTCCGCACTACTACGCTGACATCTTTAGCGTAATGGCAAGTGAGGGCATTGATCTTGAGTTCGAAGACTCTGGTCTGATGCCTGACACCAAGGACGTAATCCGCATCCTGCAAGCGCGTATCTATGAGCAATTAACGATTGACCTCTGGGAAGACGCAGAAGACTTGCTCAATGAATACTT‑‑‑‑‑‑‑G‑‑‑‑‑‑‑‑‑GAGGActgtctcttatacacatctccgag                                                                                                                                                                                                                                                                                                           >  1:393233/1‑277`  
                             `agacagGCTGAAAGAAAACATCCGTTATGATGACATCCGTGACACTGATGACCTGCACGATGCTATTCACATGGCTGCCGATAATGCAGTTCCGCACTACTACGCTGACATCTTTAGCGTGATGGCAAGTGAGGGCATTGACCTTGAGTTCGAAGACTCTGGTCTGATGCCTGACACCAAGGACGTAATCCGCATCCTGCAAGCGCGTATCTATGAGCAATTAACGATTGACCTCTGGGAAGACGCAGAAGACTTGCTCAATGAATACTT‑‑‑‑‑‑‑G‑‑‑‑‑‑‑‑‑GAGGAA‑‑‑‑‑‑‑‑‑GTCG‑‑‑‑‑‑‑C‑‑ACGACAGAAAGAAATTGAC                                                                                                                                                                                                                                                                                        <  1:475498/295‑1`  
                             `aaagagacaGAAAGAAAACATCCGTTATGATGACAGCCGTGACACTGATGACCTGCACGATGCTATTCACATGGCTGCCGATAATGCAGTTCCGCACGACTACGCTGACATCTTGAGCGTAATGGCAAGTGAGGGCATTGACCTTGAGTTCGAAGACTCTGGTCTGATGCCTGACACCAAGGACGTAATCCGCATCCTGAAAGCGCGTATCTATGAGCAATTAACGATTGACCTCTGGGAAGACGCAGAAGACTTGCTCAATGAATACTT‑‑‑‑‑‑‑G‑‑‑‑‑‑‑‑‑GAGGAA‑‑‑‑‑‑‑‑‑GTCG‑‑‑‑‑‑‑C‑‑ACGACAGAAAGAAATTGAC                                                                                                                                                                                                                                                                                        <  1:93941/292‑1`  
                                   `GCTGAAAGAAAACATCCGTTATGATGACATCCGTGACACTGATGACCTGCACGATGCTATTCACATGGCTGCCGATAATGCAGTTCCGAACTACTACGCTGACATCTTTAGCGTAATTGCAAGTGAGGGCATTGACCTTGAGTTCGAAGACTCTGGTCTGATGCCTGACACCAATGACGTAATCCGCATCCTTCAAGCGCGTATCTATGAGCAATTACCGATTGACCTCTGGTAAGACGCAGAAGACTTGCTCAATGAATACTT‑‑‑‑‑‑‑G‑‑‑‑‑‑‑‑‑GAGGAA‑‑‑‑‑‑‑‑‑GTCG‑‑‑‑‑‑‑C‑‑ACGACCGAACGAAATTGACCGCGCT                                                                                                                                                                                                                                                                                  >  1:146762/1‑301`  
                                       `AAAGAAAACATCCGTTATGATGACATCCGTGACACTGATGACCTGCACGATGCTATTCACATGGCTGCCGATAATGCAGTTCCGCACTACTACGCTGACATCTTTAGCGTAATGGCAAGTGAGGGCATTGACCTTGAGTTCGAAGACTCTGGTCTGATGCCTGACACCAAGGACGTAATCCGCATCCTGCAAGCGCGTATCTATGAGCAATTAACGATTGACCTCTGGGAAGACGCAGAAGACTTGCTCAATGAATACTT‑‑‑‑‑‑‑G‑‑‑‑‑‑‑‑‑GAGGAA‑‑‑‑‑‑‑‑‑GTCG‑‑‑‑‑‑‑C‑‑ACGACAGAAAGAAATTGACCGCGCTAAGG                                                                                                                                                                                                                                                                              <  1:187733/301‑1`  
                                            `AAACATCCGTTATGTTGACATCCGTGACACTGATGACCTGCATGATGCTATTCACATGGCTGCCGATAATGCAGTTCCGCACTACTACGCTGACATCTTTAGCGTAATGGCAAGTGAGGGCATTGACCTTGAGTTCGAAGACTCTGGTCTGATGCCTGACACCAAGGACGTAATCCGCATCCTGCAAGCGCGTATCTATGAGCAATTAACGATTGACCTCTGGGAAGACGCAGAAGACTTGCTCAATGAATACTT‑‑‑‑‑‑‑G‑‑‑‑‑‑‑‑‑GAGGAA‑‑‑‑‑‑‑‑‑GTCG‑‑‑‑‑‑‑C‑‑ACGACAGAAAGAAATTGACCGCGCTAAGGCCCGT                                                                                                                                                                                                                                                                         >  1:669121/1‑301`  
                                                        `aGATGACATCCGTGACACTGATGACCTGCACGATGCTATTCACATGGCTGCCGATAATGCAGTTCCGCACTACTACACTGACATCTTTAGCGTAATGGCAAGTGAGGGCATTGACCTTGAGTTCGAAGACTCTGGTCTGATGCCTGACACCAAGGACGTAATCCGCATCCTGCAAGCGCGTATCTATGAGCAATTAACGATTGACCTCTGGGAAGACGCAGAAGACTTGCTCAATGAATACTT‑‑‑‑‑‑‑G‑‑‑‑‑‑‑‑‑GAGGAA‑‑‑‑‑‑‑‑‑GTCG‑‑‑‑‑‑‑C‑‑ACGACAGAAAGAAATTGACCGCGCTAAGGCCCGTAAAGAACGTCAC                                                                                                                                                                                                                                                             <  1:270547/300‑1`  
                                                         `GATGACATCCGTGACACTGATGACCTGCACGATGCTATTCACATGGCTGCCGATAATGCAGTTCCGCACTACTACGCTGACATCTTTAGCGTAATGGCAAGTGAGGGCATTGACCTTGAGTTCGAAGACTCTGGTCTGATGCCTGACACCAAGGACGTAATCCGCATCCTGCAAGCGCGTATCTATGAGCAATTAACGATTGACATCTGGGAAGACGCAGAAGACTTGCTCAATGAATACTT‑‑‑‑‑‑‑G‑‑‑‑‑‑‑‑‑GAGGAA‑‑‑‑‑‑‑‑‑GTCG‑‑‑‑‑‑‑C‑‑ACGACAGAAAGAACTTGACCGCGCTAAGGCCCGTAAAGAACGTAACG                                                                                                                                                                                                                                                            >  1:530709/1‑301`  
                                                           `TGACATCCGTGACACTGATGACCTGCACGATGCTATTCACATGGCTGCCGATAATGCAGTTCCGCACTACTACGCTGACATCTTTAGCGTAATGGCAAGTGAGGGCATTGACCTTGAGTTCGAAGACTCTGGTCTGATGCCTGACACCAAGGACGTAATCCGCATCCTGCAAGCGCGTATCTATGAGCAATTAACGATTGACCTCTGGGAAGACGCAGAAGACTTGCTCAATGAATACTT‑‑‑‑‑‑‑G‑‑‑‑‑‑‑‑‑GAGGAA‑‑‑‑‑‑‑‑‑GTCG‑‑‑‑‑‑‑C‑‑ACGACAGAAAGAAATTGACCGCGCTAAGGCCCGTAAAGAACGTCACGAG                                                                                                                                                                                                                                                          >  1:597383/1‑301`  
                                                              `CATCCGTGACACTGATGACCTGCACGATGCTATTCACAGGGCTGCCGATAATGCAGTTCCGCACTACTACGCTGACATCTTTAGCGTAATGGCAAGTGAGGGCATTGACCTTGAGTTCGAAGACTCTGGTCTGATTCCTGACACCAAGGACGTAATCCGCATCCTGCAAGCGCGTATCTATGAGCAATTAACGATTGACCTCTGGGAAGACGCAGAAGACTTGCTCAATGAATACTT‑‑‑‑‑‑‑G‑‑‑‑‑‑‑‑‑GAGGAA‑‑‑‑‑‑‑‑‑GTCG‑‑‑‑‑‑‑C‑‑ACGACAGAAAGAAATTGACCGCGCTAAGGCCCGTAAAGAACGTCACGAGGGG                                                                                                                                                                                                                                                       <  1:418652/301‑1`  
                                                                     `ggatgtgtataagagacaGATGCGATTCACATGGCTGCCGATAATGCAGTTCCGCACTACTACGCTGACATCTTTAGCGTAATGGCAAGTGAGGGCATTGACCTTGAGTTCGAAGACTCTGGTCTGATGCCTGACACCAAGGACGTAATCCGCATCCTGCAAGCGCGTATCTATGAGCAATTAACGATTGACCTCTGGGAAGACGCAGAAGACTTGCTCAATGAATACTT‑‑‑‑‑‑‑G‑‑‑‑‑‑‑‑‑GAGGAA‑‑‑‑‑‑‑‑‑GTCG‑‑‑‑‑‑‑C‑‑ACGACAGAAAGAAATTGACCGCGCTAAGGCCCGTAAAGAACGTCACGAGGGGCGCTTAG                                                                                                                                                                                                                                                <  1:456953/283‑1`  
                                                                      `ACACTGATGACCTGCACGATGCTATTCACATGGCTGCCGATAATGCAGTTCCGCACTACTACGCTGACATCTTTAGCGTAATGGCAAGTGAGGGCATTGACTTTGAGTTCGAAGACTCTGGTCTGATGCCTGACACCAAGGACGTAATCCGCATCCTGCAAGCGCGTATCTATGAGCAATTAACGATTGACCTCTGGGAAGACGCAGAAGCCTTGCTCAATGAATACTT‑‑‑‑‑‑‑G‑‑‑‑‑‑‑‑‑GAGGAA‑‑‑‑‑‑‑‑‑GTCG‑‑‑‑‑‑‑C‑‑ACGACAGAAAGAAATTGACCGCGATAAGGCCCGTAAAGCACGTCACGAGGGGCGCTTAGA                                                                                                                                                                                                                                               >  1:100235/1‑301`  
                                                                         `CTGATGACCTGCACGATGCTATTCACATGGCTGCCGATAATGCAGTTCCGCACTACTACGCTGACATCTTTAGCGTAATGGCAAGTGAGGGCATTGACCTTGGGTTCGAAGACTCTGGTCTGATGCCTGACCCCAAGGACGAAATCCGCATCCTTCAAGCGCGTATCTATGAGCAATTATCGATTGACCTCTGGGACGACGCAGACGACTTGCTCAATGAATACTT‑‑‑‑‑‑‑G‑‑‑‑‑‑‑‑‑GGGGAA‑‑‑‑‑‑‑‑‑GTCG‑‑‑‑‑‑‑C‑‑ACGCCAGAAAGCATGTGAGCGTGTTAAGCCCGGTAAAGAtgtctcttatacaaatctcggagc                                                                                                                                                                                                                                            >  1:359415/1‑277`  
                                                                         `CTGATGACCTGCACGATGCTATTCACATGGCTGCCGATAATGCAGTTCCGCACTACTACGCTGACATCTTTAGCGTAATGGCAAGTGAGGGCATTGACCTTGAGTTCGAAGACTCTGGTCTGATGCCTGACACCAAGGACGTAATCCGCATCCTGCAAGCGCGTATCTATGAGCAATTAACGATTGACCTCTGGGAAGACGCAGAAGACTTGCTCAATGAATACTT‑‑‑‑‑‑‑GGAGGAAGTCGAGGAA‑‑‑‑‑‑‑‑‑GTCG‑‑‑‑‑‑‑C‑‑ACGACAGAAAGAAATTGACCGCGCTAAGGCCCGTAAAGAACctgtctcttatac                                                                                                                                                                                                                                                     >  1:413427/1‑288`  
                                                                             `acACCTGCACGATGCTATTCAAATGGCTGACGAGAATGCAGTTCCGCACAAATACGCTGACATCTTTAGCGTAATGGCAAGTGAGGGCATTGACCTTGAGTTCGAAGACTCTGGTCTGATGCCTGACACCAAGGACGTAATCCGCATCCTGCAAGCGCGTATCTATGAGCAATTAACGATTGACCTCTGGGAAGACGCAGAAGACTTGCTCAATGAATACTT‑‑‑‑‑‑‑G‑‑‑‑‑‑‑‑‑GAGGAA‑‑‑‑‑‑‑‑‑GTCG‑‑‑‑‑‑‑C‑‑ACGACAGAAAGAAATTGACCGCGCTAAGGCCCGTAAAGAACGTCACGAGGGGCGCTTAGAGGCACGC                                                                                                                                                                                                                                        <  1:538252/299‑1`  
                                                                                               `TCACATGGCTGCCGATAATGCAGTTCCGCACTACTACGCTGACATCTTTAGCGTAATGGCAAGTGAGGGCATTGACCTTGAGTTCGAAGACTCTGGTCTGATGCCTGACACCAAGGACGTAATCCGCATCCTGCAATCTCGTATCTATGAGCAATTAACGATTGACCTCTGGGAAGACGCAGAAGACTTGCTCAATGCATACTT‑‑‑‑‑‑‑G‑‑‑‑‑‑‑‑‑GAGGAA‑‑‑‑‑‑‑‑‑GTCG‑‑‑‑‑‑‑C‑‑ACGACAGAAAGAAGTTGACCGCGCTAAGGCCCGTAAAGAACGTCACGAGGGGCGCTTAGAGGCACGCAGCGTGAAACGACGCAAa                                                                                                                                                                                                                      >  1:633991/1‑300`  
                                                                                                             `ATAATGCAGTTCCGCACTACTACGCTGACATCTTTAGCGTAATGGCAAGTGAGGGCATTGACCTTGAGTTCGAAGACTCTGGTCTGATGCCTGACACCACGGACGTAATACGCATCCTGCAAGCGCGTATCTATGAGCAATTAACGATTGACCTCTGGGAAGACGCAGAAGACTTGCTCAATGAATACTT‑‑‑‑‑‑‑G‑‑‑‑‑‑‑‑‑GAGGAC‑‑‑‑‑‑‑‑‑GTCG‑‑‑‑‑‑‑C‑‑ACGACAGAAAGAAATTGACCGCGCTCACGCCATTAAAGAACGTCACGAGGGGAGCTTAGAGGCACGCAGAGTCAAACGTCGCAACCCACAGACACGTAA                                                                                                                                                                                                        >  1:481273/1‑301`  
                                                                                                             `ATAATGCAGTTCCGCACTACTACGCTGACATCTTTAGCGTAATGGCAAGTGAGGGCATTGACCTTGAGTTCGAAGACTCTGGTCTGATGCCTGACACCAAGGACGTAATCCGCATCCTGCAAGCGCGTATCTATGAGCAATTAACGATTGACCTCTGGGAAGATGCAGAAGACTTGCTCAATGAATACTT‑‑‑‑‑‑‑G‑‑‑‑‑‑‑‑‑GAGGAA‑‑‑‑‑‑‑‑‑GTCG‑‑‑‑‑‑‑C‑‑ACGACAGAAAGAAATTGACCGCGCTAAGGCCCGTAAAGAACGTCACGAGGGGCGCTTAGAGGCACGCAGATTCAAACGTCGCAACCGCAAGGCACGTAA                                                                                                                                                                                                        <  1:293708/301‑1`  
                                                                                                                     `GTTCCGCACTACTACGCTGACATCTTTAGCGTAATGGCAAGTGAGGGCATTGACCTTGAGTTCGAAGACTCTGGTCTGATGCCTGACACCAAGGACGTAATCCGCATCCTGCAAGCGCGTATCTATGAGCAATTAACGATTGACCTCTGGGAAGACGCAGAAGACTTGCTCAATGAATACTT‑‑‑‑‑‑‑GGAGGAAGTCGAGGAA‑‑‑‑‑‑‑‑‑GTCG‑‑‑‑‑‑‑C‑‑ACGACAGAAAGAAATTGACCGCGCTAAGGCCCGTAAAGAACGTCACGAGGGGCGCTTAGAGGCACGCAGATTCAAACGTCGCAACCGCAAGGCACGTA                                                                                                                                                                                                         >  1:257317/1‑301`  
                                                                                                                       `TCCGCACTACTACGCTGACATCTTTAGCGTAATGGCAAGTGAGGGCATTGACCTTGAGTTCGAAGACTCTGGTCTGATGCCTGACACCAAGGACGTAATCCGCATCCTGCAAGCGCGTATCTATGAGCAATTAACGATTGACCTCTGGGAAGACGCAGAAGACTTGCTCAATGAATACTT‑‑‑‑‑‑‑G‑‑‑‑‑‑‑‑‑GAGGAA‑‑‑‑‑‑‑‑‑GTCG‑‑‑‑‑‑‑C‑‑ACGACAGAAAGAAATTGACCGCGCTAAGGCCCGTAAAGAACGTCACGATGGGCGCTTAGAGGCACGCAGATTCAAACGTCGCAACCGCAAGGCACGTAAGGCACACAAA                                                                                                                                                                                              >  1:435301/1‑301`  
                                                                                                                         `acagCTACTACGCGGACATTTGTAGCGTAATGGCAAGTGAGGGCATTGACCTTGAGTTCGAAGACTCTGGTCTGATGCCTGACACCAAGGACGTAATCCGCATCCTGCAAGCGCGTATCTATGAGCAATTAACGATTGACCGCTGGGAAGACGCAGAAGACTTGCTCAATGAATACTT‑‑‑‑‑‑‑G‑‑‑‑‑‑‑‑‑GAGGAA‑‑‑‑‑‑‑‑‑GTCG‑‑‑‑‑‑‑C‑‑ACGACAGAAAGAAATTGACCGCGCTAAGGCCCGTAAAGAACGTCACGAGGGGCGCTTAGAGGCACGCAGATTCAAACGTCGCAACCGCAAGGCACGTAAAGCACACAAAGC                                                                                                                                                                                            <  1:76140/297‑1`  
                                                                                                                          `aagagacatcgttgccacCTTTAGCGTAATGGCAAGTGAGGGCATTGACCTTTAGTTCGAAGACTCTGGTCTGATGCCTGACACCAAGGACGTAATCCGCATCCTGCAAGCGCGTATCTATGAGCAATTAACGATTGACCTCTGGGAAGACGCAGAAGACTTGCTCAATGAATACTT‑‑‑‑‑‑‑G‑‑‑‑‑‑‑‑‑GAGGAA‑‑‑‑‑‑‑‑‑GTCG‑‑‑‑‑‑‑C‑‑ACGAAAGAAAGAAATTGACCGCGCTAAGGCCCGTAAAGAACGTCACGAGGGGCGCTTAGAGGCACGCAGATTCAAACGTCGCAACCGAAAGGCACGTAAAGCACACTAAGCT                                                                                                                                                                                           <  1:70160/283‑1`  
                                                                                                                             `CTACTACGCTGACATCTTTAGCGTAATGGCAAGTGAGGGCATTGACCTTGAGTTCGAAGACTCTGGTCTGATGCCTGACACCAAGGACGTAATCCGCATCCTGCAAGCGCGTATCTATGAGCAATTAACGATTGACCTCTGGGAAGACGCAGAAGACTTGCTCAATGAATACTT‑‑‑‑‑‑‑G‑‑‑‑‑‑‑‑‑GAGGAA‑‑‑‑‑‑‑‑‑GTCG‑‑‑‑‑‑‑C‑‑ACGACAGAAAGAAATTGACCGCGCTAAGGCCCGTAAAGAACGTCACGAGGGGCGCTTAGAGGCACGCAGATTCAAACGTTGCAACCGCAAGGCACGTAAAGCACACAAAGCTAAG                                                                                                                                                                                        >  1:288258/1‑301`  
                                                                                                                             `CTACTACGCTGACATCTTTAGCGTAATGGCAAGTGAGGGCATTGACCTTGAGTTCGAAGACTCTGGTCTGATGCCTGACACCAAGGACGTAATCCGCATCCTGCAAGCGCGTATCTATGAGCAATTAACGATTGACCTCTGGGAAGACGCAGAAGACTTGCTCAATGAATACTT‑‑‑‑‑‑‑G‑‑‑‑‑‑‑‑‑GAGGAA‑‑‑‑‑‑‑‑‑GTCG‑‑‑‑‑‑‑C‑‑ACGACAGAAAGAAATTGACCGCGCTAAGGCCCGTAAAGAACGTCACGAGGGGCGCTTAGAGGCACGCAGATTCAAACGTCGCAACCGCCAGGCACGTAAAGCACACAAAGATAAG                                                                                                                                                                                        >  1:369260/1‑301`  
                                                                                                                                  `ACGCTGACATCTTTAGCGTAATGGCAAGTGAGGGCATTGACCTTGAGTTCGAAGACTCTGGTCTGATGCCTGACACCAAGGACGTAATCCGCATCCTGCAAGCGCGTATCTATGAGCAATTAACGATTGACCTCTGGGAAGACGCAGAAGACTTGCTCAATGAATACTT‑‑‑‑‑‑‑G‑‑‑‑‑‑‑‑‑GAGGAA‑‑‑‑‑‑‑‑‑GTCG‑‑‑‑‑‑‑C‑‑ACGACAGAAAGAAATTGACCGCGCTAAGGCCCGTAAAGAACGTCACGAGGGGCGCTTAGAGGCACGCAGATTCAAACGTCGCAACCGCAAGGCACGTAAAGCACACAAAGCTAAGCGCGA                                                                                                                                                                                   >  1:451068/1‑301`  
                                                                                                                                     `catACAGCTTGAGCGTAATGGCAAGGGAGGTCATTTACCTTGAGTTCGACGCGTCGGGGCTGGTGCCTTACACCAAGGACGTAATCCGCATCCTGCAAGCGCGTATTTATGAGCAATTAACGATTGACCTATGGGATGACGCAGAAGACTTGCTCAATGAATACTG‑‑‑‑‑‑‑G‑‑‑‑‑‑‑‑‑GAGGAA‑‑‑‑‑‑‑‑‑GTCG‑‑‑‑‑‑‑C‑‑CCGACAGAAAGAAATTTCCCGCGCTAAGGCCCGTAAAGAACGTCACGAGGGGCGCTTAGAGGCACGCAGATTCAAACGTCGCAACCGCAAGGCACGTAAAGCACACAAAGCTAAGCGCGAAAG                                                                                                                                                                                <  1:51143/298‑1`  
                                                                                                                                     `CTGACATCTTTAGCGTAATGGCAAGTGAGGGCATTGACCTTGAGTTCGAAGACTCTGGTCTGATGCCTGACACCAAGGACGTAATCCGCATCCTGCAAGCGCGTATCTATGAGCAATTAACGATTGACCTCTTGGAAGACGCAGAAGACTTGCTCAATGAATACTT‑‑‑‑‑‑‑G‑‑‑‑‑‑‑‑‑GAGGAA‑‑‑‑‑‑‑‑‑GTCG‑‑‑‑‑‑‑C‑‑ACGACAGAAAGAAATTGACCGCGCTAAGGCCCGTAAAGAACGTCACGAGGGGCGCTTAGAGGCACGCAGATTCAAACGTCGCAACCGCAAGGCACGTAAAGCACACAAAGCTAAGCGCGAAAG                                                                                                                                                                                >  1:524712/1‑301`  
                                                                                                                                     `CTGACATCTTTAGCGTAATGGCAAGTGAGGGCATTGACCTTGAGTTCGAAGACTCTGGTCTGATGCCTGACACCAAGGACGTAATCCGCATCCTGCAAGCGCGTATCTATGAGCAATTAACGATTGACCTATGGGAAGACGCAGAAGACTTGCTCAATGAATACTT‑‑‑‑‑‑‑GGAGGAAGTCGAGGAA‑‑‑‑‑‑‑‑‑GTCG‑‑‑‑‑‑‑C‑‑ACGACAGAAAGAAATTGACCGCGCTAAGGCCCGTAAAGAACGTCACGAGGGGCGCTTAGAGGCACGCAGATTCAAACGTCGCAACCGCAAGGCACGTAAAGCACACAAAGCTAA                                                                                                                                                                                         >  1:462326/1‑301`  
                                                                                                                                            `CTTTAGCGTAATGGCAAGTGAGGGCATTGACCTTGAGTTCGAAGACTCTGGTCTGATGCCTGACACCAAGGACGTAATCCGCATCCTGCAAGCGCGTATCTATGAGCAATTAACGATTGACCTCTGGGAAGACGCAGAAGACTTGCTCAATGAATACTT‑‑‑‑‑‑‑G‑‑‑‑‑‑‑‑‑GAGGAA‑‑‑‑‑‑‑‑‑GTCG‑‑‑‑‑‑‑C‑‑ACGACAGAAAGAAATTGACCGCGCTAAGGCCCGTAAAGAACGTCACGAGGGGCGCTTAGAGGCACGCAGATTCAAACGTCGCAACCGCAAGGCACGTAAAGCACACAAAGCTAAGCGCGAAAGAATGCTT                                                                                                                                                                         >  1:664508/1‑301`  
                                                                                                                                              `TTAGCGTAATGGCAAGTGAGTGCATTGACCTTGAGTTCGAAGACTCTGGTCTGATGCCTGACACCAAGGACGTAATCCGCATCCTGCAAGCGCGTATCTATGAGCAATTAACGATTGACCTCTGGGAAGACGCAGAAGACTTGCTCAATGAATACTT‑‑‑‑‑‑‑GGAGGAAGTCGAGGAA‑‑‑‑‑‑‑‑‑GTCG‑‑‑‑‑‑‑C‑‑ACGACAGAAAGAAATTGACCGCGCTAAGGCCCGTAAAGAACGTCACGAGGGGCGCTTAGAGGCACGCAGATTCAAACGTCGCAACCGCAAGGCACGTAAAGCACACAAAGCTAAGCGCGAAAG                                                                                                                                                                                <  1:623675/301‑1`  
                                                                                                                                                 `GCGTAATGGCAAGTGAGGGCATTGACCTTGAGTTCGAAGACTCTGGTCTGATGCCTGACACCAAGGACGTAATCCGCATCCTGCAAGCGCGTATCTATGAGCAATTAACGATTGACCTCTGGGAAGACGCAGAAGACTTGCTCAATGAATACTT‑‑‑‑‑‑‑GGAGGAAGTCGAGGAA‑‑‑‑‑‑‑‑‑GTCG‑‑‑‑‑‑‑C‑‑ACGACAGAAAGAAATTGACCGCGCCAAGGCCCGTAAAGAACGTCACGAGGGGCGCTTAGAGGCACGCAGATTCAAACGTCGCAACCGCAAGGCCCGTAAAGCACACAAAGCTAAGCGCGAAAGAAg                                                                                                                                                                             >  1:363557/1‑300`  
                                                                                                                                                   `GTAATGGCAAGTGAGGGCATTGACCTTGAGTTCGAAGACTCTGGTCTGATGCCTGACACCAAGGACGTAATCCGCATCCTGCAAGCGCGTATCTATGAGCAATTAACGATTGACCTCTGGGAAGACGCAGAAGACTTGCTCAATGAATACTT‑‑‑‑‑‑‑G‑‑‑‑‑‑‑‑‑GAGGAA‑‑‑‑‑‑‑‑‑GTCG‑‑‑‑‑‑‑C‑‑ACGACAGAAAGAAATCGACCGCGCTAAGGCCCGTAAAGAACGTCACGAGGGGCGCTTAGAGGCACGCAGATTCAAACGTCGCAACCGCAAGGCACGTAAAGCACACAAAGCTAAGCACGAAAGAATGCTTGCTGCGg                                                                                                                                                                  >  1:551536/1‑300`  
                                                                                                                                                      `ATGGCAAGTGAGGGCATTGACCTTGAGTTCGAAGACTCTGGTCTGATGCCTGACACCAAGGACGTAATCCGCATCCTGCAAGCGCGTATCTATGAGCAATTAACGATTGACCTCTGGGAAGACGCAGAAGACTTGCTCAATGAATACTT‑‑‑‑‑‑‑G‑‑‑‑‑‑‑‑‑GAGGAA‑‑‑‑‑‑‑‑‑GTCG‑‑‑‑‑‑‑C‑‑ACGACAGAAAGAAATTGACCGCGCTAAGGCCCGTAAAGAACGTCACGAGGGGCGCTTAGAGGCACGCAGATTCAAACGTCGCAACCGCAAGGCACGTAAAGAACACAAAGCTAAGCGCGAAAGAATGCTTGCTGCGTGGC                                                                                                                                                               >  1:182996/1‑301`  
                                                                                                                                                      `ATGGCAAGTGAGGGCATTGACCTTGAGTTCGAAGACTCTGGTCTGATGCCTGACACCAAGGACGTAATCCGCATCCTGCAAGCGCGTATCTATGAGCAATTAACGATTGACCTCTGGGAAGACGCAGAAGACTTGCTCAATGAATACTT‑‑‑‑‑‑‑G‑‑‑‑‑‑‑‑‑GAGGAA‑‑‑‑‑‑‑‑‑GTCG‑‑‑‑‑‑‑C‑‑ACGACAGAAAGAAATTGACCGCGCTAAGGCCCGTAAAGAACGTCACGAGGGGCGCTTAGAGGCACGCAGATTCAAACGTCGCAACCGCAAGGCACGTAAACCACACACAGCTAAGCGCGACAGAATGCTTGCTGCGGGGC                                                                                                                                                               >  1:199263/1‑301`  
                                                                                                                                                        `GGCAAGTGAGGGCATGGAACTTGGGTTCGAAGCCTCTGGTCTGATGCCTGACACCAAGGACGTAATCCGCATCCTGCAAGCGCGTATCTATGAGCAATTAACGATTGACCGCTGGGAAGACGCAGAAGACTTGCTCAATGAATACTT‑‑‑‑‑‑‑GGAGGAAGTCGAGGAA‑‑‑‑‑‑‑‑‑GTCG‑‑‑‑‑‑‑C‑‑ACGACAGAAAGAAATTGACCGCGCTAAGGCCCGTAAAGAACGTCACGAGGGGCGCTTAGAGGCACGCAGATTCAAACGTCGCAACCGCAAGGCACGTAAAGCACACAAAGCTAAGCGCGAAAGAATGCTTGCT                                                                                                                                                                      <  1:221193/301‑1`  
                                                                                                                                                           `agggGAGGCCATTGACCTTGAGTTCGAATACTCTGGTCTGATGCCTGACACCAAGGACGTAATCCGCATCCTGCAAGCGCGTATCTATGAGCAATTAACGATTGACCGCTGGGAAGACGCAGAAGACTTGCTCAATGAATACTT‑‑‑‑‑‑‑GTAGGAAGTCGAGGAA‑‑‑‑‑‑‑‑‑GTCG‑‑‑‑‑‑‑C‑‑ACGACAGAAAGAAATTGACCGCGCTAAGGCCCGTAAAGAACGTCACGAGGGGCGCTTAGAGGCACGCAGATTCAAACGTCGCAACCGCAAGGCACGTAAAGCACACAAAGCTAAGCGCGAAAGAATGCTTGCTGCG                                                                                                                                                                   <  1:654712/297‑1`  
                                                                                                                                                             `GTGAGTGCATTGACCTTGAGTTCGAAGACTCTGGTCTGATGCCTGACACCAAGGACGTAATCCGCATCCTGCAAGCGCGTATCTATGAGCAATTAACGATTGACCTCTGGGAAGACGCAGAAGACTTGCTCAATGAATACTT‑‑‑‑‑‑‑GGAGGAAGTCGAGGAA‑‑‑‑‑‑‑‑‑GTCG‑‑‑‑‑‑‑C‑‑ACGACAGAAAGAAATTGACCGCGCTAAGGCCCGTAAAGAACGTCACGAGGGGCGCTTAGAGGCACGCAGGTTCAAACGTCGCAACCGCAAGGCACGTAAAGCACACAAACCTAAGCGCGACAGAATGCTTGCTTCGTG                                                                                                                                                                 >  1:564143/1‑301`  
                                                                                                                                                                           `ggctcggagatgtgtataagagacagGCCTGACACCAAGGACGTAATCCGCATCCTGCAAGCGCGTATCTATGAGCAATTAACGATTGACCTCTGGGAAGACGCAGAAGACTTGCTCAATGAATACTT‑‑‑‑‑‑‑GGAGGAAGTCGAGGAA‑‑‑‑‑‑‑‑‑GTCG‑‑‑‑‑‑‑C‑‑ACGACAGAAAGAAATTGACCGCGCTAAGGCCCGTAAATAACGTCACGAGGGGCGCTTAGAGGCACGCAGATTCAAACGTCGCAACCGCAAGGCACGTAAAGCACACAAAGCTAAGCGCGAAAGAATGCTTGCTGCGTGGCGATGGGCTGAAC                                                                                                                                                   <  1:170893/275‑1`  
                                                                                                                                                                                   `CGAAGACTCTGTTCGGATGCCTGAAAACAAGGACTGACTCCGCAGCCTGCAAGAGCGTATCTATGAGCAATTAACGATTGACCTCTGGGAGGACGAAGAAGACTTGCTCAATGAATACTT‑‑‑‑‑‑‑G‑‑‑‑‑‑‑‑‑GAGGAAGTCGAGGACGTCG‑‑‑‑‑‑‑C‑‑ACGACAGAAAGAAATTGACCGCGCTAAGGCACGTAAAGAACGCCACGAGGGGCGCTTAGAGGCACGCAGATTCAAACGTCGCAACCGCAAGGCACGTAAAGCACACAAAGCTAAGCGCGAAAGAATGCTTGCTGCGTGGCGATGGGCTGAACGTCAAGAA                                                                                                                                           <  1:176591/301‑1`  
                                                                                                                                                                                   `CGAAGACTCTGGTCTGATGACTGACACCAAGGACGTAATCCGCATCCTGCAAGCGCGTATCTATGAGCAATTAACGATTGACCTCTGGGAAGACGCAGAAGACTTGCTCAATGAATACTT‑‑‑‑‑‑‑G‑‑‑‑‑‑‑‑‑GAGGAA‑‑‑‑‑‑‑‑‑GTCG‑‑‑‑‑‑‑C‑‑ACGACAGAAAGAAATTGACCGCGCTAAGGCCCGTAAAGAACGTCACGAGGGGCGCTTAGAGGCACGCAGATTCAAACGTCGCAACCGCAAGGCACGTAAAGCACACAAAGCTAAGCGCGAAAGAATGCTTGCTGCGTGGCGATGGGCTGAACGTCAAGAACGGCGTAAC                                                                                                                                  <  1:494436/301‑1`  
                                                                                                                                                                                     `AAGACTTTGGTCTGATGCCTGACACCAAGGACGTAATCCGCATCCTGCAAGCGCGTATCTATGAGCAATTAACGATTGACCTCTGGGAAGACGCAGAAGACTTGCTCAATGAATACTT‑‑‑‑‑‑‑G‑‑‑‑‑‑‑‑‑GAGGAA‑‑‑‑‑‑‑‑‑GTCG‑‑‑‑‑‑‑C‑‑ACGACAGAAAGAAATTGACCGCGCTAAGGCCCGTAAAGAACGTCACGAGGGGCGCTTAGAGGCACGCAGATTCAAACGTCGCAACCGCAAGGCACGTAAAGCACACAAAGCTAAGCGCGAAAGAATGATTGCTGCGTGGCGATGGGCTGAACGTCAAGAACGGCGTAACCA                                                                                                                                <  1:28070/301‑1`  
                                                                                                                                                                                     `AAGACGCTGGTCTGATGCCGGAAACCAAGAAGGTAATCCGCATGCTGCAAGGGCGTATCTATGAGCAATTAACGATTGACCTCTGGGAAGACGCAGAAGACTTGCTCAATGAATACTT‑‑‑‑‑‑‑G‑‑‑‑‑‑‑‑‑GAGGAA‑‑‑‑‑‑‑‑‑GTCG‑‑‑‑‑‑‑C‑‑ACGACAGAAAGAAATTGACCGCGCTAAGGCCCGTAAAGAACGTCACGAGGGGCGCTTAGAGGCACGCAGATTCAAACGTCGCAACCGCAAGGCACGTAAAGCACACAAAGCTAAGCGCGAAAGAATGCTTGCTGCGTGGCGATGGGCTGAACGTCAAGAACGGCGTAACCA                                                                                                                                <  1:385568/301‑1`  
                                                                                                                                                                                       `GACTCTGGTCTGATGCCTGACACCAAGGACGTAATCCGCATCCTGCAAGCGCGTATCTATGAGCAATTAACGATTGACCTCTGGGAAGACGCAGAAGACTTGCTCAATGAATACTT‑‑‑‑‑‑‑G‑‑‑‑‑‑‑‑‑GAGGAA‑‑‑‑‑‑‑‑‑GTCG‑‑‑‑‑‑‑C‑‑ACGACAGAAAGAAATTGACCGCGCTAAGGCCCGTAAAGAACGTCACGAGGGGCGCTTAGAGGCACGCAGATTCAAACGTCGCAACCGCAAGGCACGTAAAGCACACAAAGCTAAGCGCGAAAGAATGCTTGCTGCGTGGCGATGGGCTGAACGTCAAGAACGGCGTAACCATG                                                                                                                              >  1:488309/1‑301`  
                                                                                                                                                                                        `ACTCTGGTCTGATGCCTGACACCAAGGACGTAATCCGCATCCTGCAAGCGCGTATCTATGAGCAATTAACGATTGACCTCTGGGAAGACGCAGAAGACTTGCTCAATGAATACTT‑‑‑‑‑‑‑G‑‑‑‑‑‑‑‑‑GAGGAA‑‑‑‑‑‑‑‑‑GTCG‑‑‑‑‑‑‑C‑‑ACGACAGAAAGAAATTGACCGCGCTAAGGCCCGTAAAGAACGTCACGAGGGGCGCTTAGAGGCACGCAGATTCAAACGTCGCAACCGCAAGGCACGTAAAGCACACAAAGCTAAGCGCGAAAGAATGCTTGCTGCGTGGCGAGGGGCTGAACGTCAAGCACGGCGTAACCAGGA                                                                                                                             >  1:107319/1‑301`  
                                                                                                                                                                                          `tctggattaATGCCAGAAACCAAGGACGTAACCCGCATCCTGCAAGCGCGTATCTATGAGCAATTAACGATTGACCTCTGGGAAGACGCAGAAGACTTGCTCAATTAAGACTT‑‑‑‑‑‑‑G‑‑‑‑‑‑‑‑‑GAGGAA‑‑‑‑‑‑‑‑‑GTCG‑‑‑‑‑‑‑A‑‑AGTACAGAAATAAATTGACCGCGCTAAGGCCCGTAAAGAACGTCACGAGGGGCGCTTAGAGGCATGCAGATTCAAACGTCGCAACCGCAAGGCACGTAAAGCACCCAAAGCTAAGCGCGAAAGAATGCTTGCTGCGTGGAGATGGGCTGAACGTCAAGAACGGCGTAACCATGAGG                                                                                                                           <  1:429559/292‑1`  
                                                                                                                                                                                          `taTGGTCTGATGCCTGACACCAAGGACGTAAGCCGCATACTGTAAGCGCGTATCTATGAGCAATTAACGATTGACCTCTGGGAAGACGCAGAAGACTTGCACAATGAATAATT‑‑‑‑‑‑‑G‑‑‑‑‑‑‑‑‑GAGGAA‑‑‑‑‑‑‑‑‑GTCG‑‑‑‑‑‑‑C‑‑ACGACAGAAAGAAATGGACCGCGCTAAGGCAAGTAAAGAACGGCACGAGGGGCGCTGAGAGGCACGCAGATTCAAACGTCGCAACCGCAAGGCACGTAAAGCACACAAAGATAAGCGCGAAAGAATGCTTGCTGCGTGGCGATGGGCTGAACGGCAAGAACGGCGTAACCATGAGG                                                                                                                           <  1:647933/299‑1`  
                                                                                                                                                                                          `TCTGGTCTGATGCCTGACACCAAGGACGTAATCCGCATCCTGCAAGCGCGTATCTATGAGCAATTAACGATTGACCTCTGGGAAGACGCAGAAGACTTGCTCAATGAATACTT‑‑‑‑‑‑‑G‑‑‑‑‑‑‑‑‑GAGGAA‑‑‑‑‑‑‑‑‑GTCG‑‑‑‑‑‑‑C‑‑ACGACAGAAAGAAATTGACCGCGATAAGGCCCGTAAAGAACGTCACGAGGGGCGCTTAGAGGCACGCAGATTCAAACGTCGCAACCGCAAGGCACGTAAAGCACACAAAGCTAAGCGCGAAAGAATGCTTGCTGCGTGGCGATGGGCTGAACGTCAAGAA‑GGCGTAACTATGAGGg                                                                                                                          >  1:159251/1‑300`  
                                                                                                                                                                                           `CTGGTCTGATGCCTGAAACCAAGGACGTAATCCGCAACCTGCAAGCGCGTATCTATGAGCAATTAACGATTGACCTCTGGGAAGACGCAGAAGACTTGCTCAATGAATACTT‑‑‑‑‑‑‑G‑‑‑‑‑‑‑‑‑GAGGAA‑‑‑‑‑‑‑‑‑GTCG‑‑‑‑‑‑‑C‑‑ACGACAGAAAGAAATTGACCGCGCTAAGGCCCGTAAAGAACGTCACGAGGGGCGCTTAGAGGCACGCAGATTCAAACGTCGCAACCGCAAGGCACGTAAAGCACACAAAGCTAAGCGCGAAAGAATGCTTGCTGCGTGGCGATGGGCTGAACGTCAAGAACGGCGTAACCATGAGGT                                                                                                                          <  1:518285/301‑1`  
                                                                                                                                                                                             `GGTCTGATGCCTGACACCAAGGACGTAATCCGCATCCTGCAAGCGCGTATCTATGAGCAATTAACGATTGACCTCTGGGAGGACGCAGAAGACTTGCTCAATGAATACTT‑‑‑‑‑‑‑G‑‑‑‑‑‑‑‑‑GAGGAA‑‑‑‑‑‑‑‑‑GTCG‑‑‑‑‑‑‑C‑‑ACGACAGAAAGAAATTGACCGCGCTAAGGCCCGTAAAGAACGTCACGAGGGGCGCTTAGAGGCACGCAGATTCAAACGTCGCAACCGCAAGGCACGTAAAGCACACAAAGCTAAGCGCGAAAGAATGCTTGCTGCGTGGCGATGGGCTGAACGTCAAGAACGGCGTAACCATGAGGTct                                                                                                                        >  1:81570/1‑299`  
                                                                                                                                                                                              `GTCTGATTCCTGACACCAAGGAAGTAATCCGCATCCTGCAAGCGCGTATCTATGAGCAATTAACGATTGACCTCTGGGAAGACGCAGAAGACTTGCTCAATGAATACTT‑‑‑‑‑‑‑G‑‑‑‑‑‑‑‑‑GAGGAA‑‑‑‑‑‑‑‑‑GTCG‑‑‑‑‑‑‑C‑‑ACGACAGAAAGAAATTGACCGCGCTAAGGCCCGTAAAGAACGTCACGAGGGGCGCTTAGAGGCACGCAGATTCAAACGTCGCAACCGCAAGGCACGTAAAGCACACAAAGCTAAGCGCGAAAGAATGCTTGCTGCGTGGCGATGGGCTGAACGTCAAGCACGGCGTAACCATGAGGTAGC                                                                                                                       >  1:572158/1‑301`  
                                                                                                                                                                                                `tcggagagttgtatcagagacagAATCCGCATCCTGCAAGCGCGTATCTATGAGCAATTAACGATTGACCTCTGGGAAGACGCAGAAGACTTGCTCAATGAATACTT‑‑‑‑‑‑‑G‑‑‑‑‑‑‑‑‑GAGGAA‑‑‑‑‑‑‑‑‑GTCG‑‑‑‑‑‑‑C‑‑ACGACAGAAAGAAATTGACCGCGCTAAGGCCCGTAAAGAACGTCACGAGGGGCGCTTAGAGGCACGCAGATTCAAACGTCGCAACCGCAAGGCACGTAAAGCACACAAAGCTAAGCGCGAAAGAATGCTTGCTGCGTAGCGATGGGCTGAACGTCAAGAACGGCGTAACCATGAGGTAGCTG                                                                                                                     <  1:33594/278‑1`  
                                                                                                                                                                                                `CTGATGCCTGACACCAAGGACGTAATCCGCATCCTGCAAGCGCGTATCTATGAGCAATTAACGATTGACCTCTGGGAAGACGCAGAAGACTTGCTCAATGAATACTT‑‑‑‑‑‑‑GGAGGAAGTCGAGGAA‑‑‑‑‑‑‑‑‑GTCG‑‑‑‑‑‑‑C‑‑ACGACAGAAAGAAATTGACCGCGCTAAGGCCCGTAAAGAACGTCACGAGGGGCGCTTAGAGGCACGCAGATTCAAACGTCGCAACCGCAAGGCACGTAAAGCACACAAAGCTAAGCGCGAAAGAATGCTTGCTGCGTGGCGATGGGCTGAACGTCAAGAACGGCGTAACCATG                                                                                                                              >  1:659029/1‑301`  
                                                                                                                                                                                                  `GATGCCTGACACCAAGGACGTAATCCGCATCCTGCAAGCGCGTATCTATGAGCAATTAACGATTGACCTCTGGGAAGACGCAGAAGACTTGCTCAATGAATACTT‑‑‑‑‑‑‑GGAGGAAGTCGAGGAA‑‑‑‑‑‑‑‑‑GTCG‑‑‑‑‑‑‑C‑‑ACGACAGAAAGAAATTGACCGCGCTAAGGCCCGTAAAGAACGTCACGAGGGGCGCTTAGAGGCACGCAGATTCAAACGTCGCAACCGCAAGGCACGTAAAGCACACAAAGCTAAGCGCGAAAGAATGCTTGCTGCGTGGCGATGGGCTGAACGTCAAGAACGGCGTAACCATGAG                                                                                                                            >  1:195050/1‑301`  
                                                                                                                                                                                                     `GCCTGACACCAAGGACGTAATCCGCATCCTGCAAGCGCGTATCTATGAGCAATTAACGATTGACCTCTGGGAAGACGCAGAAGACTTGCTCAATGAATACTT‑‑‑‑‑‑‑G‑‑‑‑‑‑‑‑‑GAGGAA‑‑‑‑‑‑‑‑‑GTCG‑‑‑‑‑‑‑C‑‑ACGACAGGAAGAAATTGACCGCGCTAAGGCCCGTAAAGAACGTCACGAGGGGCGCTTAGAGGCACGCAGATTCAAACGTCGCAACCGCAAGGCACGTAAAGCACACAAAGCTAAGCGCGAAAGAATGCTTGCTGCGTGGCGATGGGCTGAACGTCAAGAACGGCGTAACCATGAGGTAGCTGTAGAT                                                                                                                >  1:275209/1‑301`  
                                                                                                                                                                                                         `aACACCAAGACAGTAATCCGCATCCTGCAAGCGCGTAGCGATGAGCAATTAACGATTGACCTCTGGGAAGACGCAGAAGACTTGCTCAATGAATACTT‑‑‑‑‑‑‑G‑‑‑‑‑‑‑‑‑GAGGAA‑‑‑‑‑‑‑‑‑GTCG‑‑‑‑‑‑‑C‑‑ACGACAGAAAGAAATTGACCGCGCTAAGGCCCGTAAAGAACGTCACGAGGGGCGCTTAGAGGCACGCAGATTCAAACGTCGCAACCGCAAGGCACGTAAAGCACACAAAGCTAAGCGCGAAAGAATGCTTGCTGCGTGGCGATGGGCTGAACGTCAAGAACGGCGTAACCATGAGGTAGCTGTAGATGTAC                                                                                                            <  1:557105/300‑1`  
                                                                                                                                                                                                            `ataagggacagAATCTGCATCCTGCAAGCGCGTATCTATGAGCAATTAACGATTGACCTCTGGGAAGACGCAGAAGACTTGCTCAATGAATACTG‑‑‑‑‑‑‑G‑‑‑‑‑‑‑‑‑GAGGAA‑‑‑‑‑‑‑‑‑GTCG‑‑‑‑‑‑‑T‑‑ACGACAGAAAGAAATTGACCGCGCTAAGGCCCGTAAAGAACGTCACGAGGGGCGCTTAGAGGCACGCAGATTCAAACGTCGCAACCGCAAGGCACGTAAAGCACACAAAGCTAAGCGCGAAAGAATGCTTGCTGCGTGGCGATGGGCTGAACGTCAAGAACGGCGTAACCATGAGGTAGCTGTAGATGTACTAG                                                                                                         <  1:590666/290‑1`  
                                                                                                                                                                                                             `CCAAGGACGTAATCCGCATCCTGCAAGCGCGTATCTATGAGCAATTAACGATTGACCTCTGGGAAGACGCAGAAGACTTGCTCAATGAATACTT‑‑‑‑‑‑‑G‑‑‑‑‑‑‑‑‑GAGGAA‑‑‑‑‑‑‑‑‑GTCG‑‑‑‑‑‑‑C‑‑ACGACAGAAAGAAATTGACCGCGCTAAGGCCCGTAAAGAACGTCACGAGGGGCGCTTAGAGGCACGCAGATTCAAACGTCGCAACCGCAAGGCACGTAAAGCACACAAAGCTAAGCGCGAAAGAATGCTTGCTGCGTGGCGATGGGCTGAACGTCAAGAACGGCGTAACCATGAGGTAGCTGTAGATGTACTAGG                                                                                                        >  1:11283/1‑301`  
                                                                                                                                                                                                              `aaagggacagATCCGCATCCTGCAAGCGCGTATCTATGAGCAATTAACGATTGACCTCTGGGAAGACGCAGAAGACTTGCTCAATGAATACTT‑‑‑‑‑‑‑G‑‑‑‑‑‑‑‑‑GAGGAA‑‑‑‑‑‑‑‑‑GTCG‑‑‑‑‑‑‑C‑‑ACGACAGAAAGAAATTGACCGCGCGAAGGCCCGTAAAGAACGTCACGAGGGGCGCTTAGAGGCACGCAGATTCAAACGTCGCAACCGCAAGGCACGTAAAGCACACAAAGCTAAGCGCGAAAGAATGCTTGCTGCGTGGCGATGGGCTGAACGTCAAGAACGGCGTAACCATGAGGTAGCTGTAGATGTACTAGGA                                                                                                       <  1:643750/291‑1`  
                                                                                                                                                                                                              `aAAGGACGTAACCCGCTTCCTGCAAGCGCTTAACTATGAGCAATTAACGATTGACCGCTGGGAAGACGCAGAAAACTTGTCCAATGAATGCTT‑‑‑‑‑‑‑G‑‑‑‑‑‑‑‑‑GAGGAA‑‑‑‑‑‑‑‑‑GTCT‑‑‑‑‑‑‑C‑‑ACGACAGAAAGAAATTGACCGCGCTAAGGGCCGTAAAGAACGGCACTAGGGGCGCTTAGAGGCACGCAGATTCAAACGTCGCAACCGCAAGGCACGTAAAGCACACAAAGCTAAGCGCGAAAGAATGCTTGCTGCGTGGCGATGGGCTGAACGTCAAGAACGGCGTAACCATGAGGTAGCTGTAGATGTACTAGGA                                                                                                       <  1:316371/300‑1`  
                                                                                                                                                                                                              `CAAGGACGTAATCCGAAACCTGCAAGCGCGGATCGATGAGCAATTAACGATTGACCTATGGGAAGACGCAGAAGACTTGCTAAATGAATACTT‑‑‑‑‑‑‑G‑‑‑‑‑‑‑‑‑GAGGAA‑‑‑‑‑‑‑‑‑GTCG‑‑‑‑‑‑‑C‑‑AAGACAGAAAGAAATTGACCGCGCTAAGGCCCGTAAAGAACGTCACGAGGGGCGCTTAGAGGCACGCAGATTCAAACGTCGCAACCGCAAGGCACGTAAAGCACACAAAGCTAAGCGCGAAAGAATGCTTGCTGCGTGGCGATGGGCTGAACGTCAAGAACGGCGTAACCATGAGGTAGCTGTAGATGTACTAGGA                                                                                                       <  1:353896/301‑1`  
                                                                                                                                                                                                                  `GACGTAATCCGCATCCTGCAAGCGCGTATCTATGAGCAATTAACGATTGACCTCTGGGAAGACTCAGAAGACTTGCTCAATGAATACTT‑‑‑‑‑‑‑G‑‑‑‑‑‑‑‑‑GAGGAA‑‑‑‑‑‑‑‑‑GTCG‑‑‑‑‑‑‑C‑‑ACGACAGAAAGAAATTGACCGCGCTAAGGCCCGTAAAGAACGTCACGAGGGGCGCTTAGAGGCACGCAGATTCAAACGTCGCAACCGCAAGGCACGTAAAGCACACAAAGCTAAGCGCGAAAGAATGCTTGCTGCGTGGCGATGGGCTGAACGTCAAGAACGGCGTAACCATGAGGctgtctcttatacacatctccgag                                                                                                   >  1:251874/1‑277`  
                                                                                                                                                                                                                   `ACGTAATCCGCATCCTGCAAGCGCGTATCTATGAGCAATTAACGATTGACCTCTGGGAAGACGCAGAAGACTTGCTCAATGAAGACTT‑‑‑‑‑‑‑GGAGGAAGTCGAGGAA‑‑‑‑‑‑‑‑‑GTCG‑‑‑‑‑‑‑C‑‑ACGACAGAAAGAAATTGACCGCGCTAAGGCCCGTAAAGAACGTCACGAGGGGCGCTTAGAGGCACGCAGATTCAAACGTCGCAACCGCAAGGCACGTAAAGCACACAAAGCTAAGCGCGAAAGAATGCTTGCTGCGTGGCGATGGGCTGAACGTCAAGAACGGCGTAACCATGAGGTAGCTGTAGATGTACT                                                                                                           <  1:348768/301‑1`  
                                                                                                                                                                                                                       `agagacagTCCTGCAAGCGCGTATCTAGGAGAAATTAACGAGTGACCTCGGAGAAGACGCAGAAGAATTGATCAATGGATACTT‑‑‑‑‑‑‑GGAGGAAGTAGAGGAA‑‑‑‑‑‑‑‑‑GTCG‑‑‑‑‑‑‑C‑‑ACAACAGAAAGAAATTAACCGCGCAAAGGCCCGTAAAGAACGTCACGAGGGGCGATTAGAGGCACGCAGATTCAAACGTCGCAACCGCAAGGCACGTAAAGCACACAAAGCTAAGCGCGAAAGAATGCTTGCTGCGTGGCTATTGGCTGAACGTCAAGAACGGCGTAACCATGAGGTAGCTGTAGATGTACTAGGA                                                                                                       <  1:228179/293‑1`  
                                                                                                                                                                                                                       `AATCCGCATCCTGCAAGCGCGTATCTATGAGCAATTAACGATTGACCTCTGGGAAGCCGCAGAAGACCTGCTCAATGAATGCTTTTAGGAAGTC‑‑‑‑‑‑‑GAGGAA‑‑‑‑‑‑‑‑‑GTCG‑‑‑‑‑‑‑C‑‑ACGACAGAAAGAAATTGACCGCGCTAAGGCCCGTAAAGAACGTCACGAGGGGCGCTTAGAGGCACGCAGATTCAAACGTCGCAACCGCAAGGCACGTAAAGCACACAAAGCTAAGCGCGAAAGAATGCTTGCTGCGTGGCGATGGGCTGAACGTCAAGAACGGCGTAACCATGAGGTAGCTGTAGATGTACTAGGA                                                                                                       <  1:578744/301‑1`  
                                                                                                                                                                                                                       `AATCCGCATCCTGCAAGCGCGTATCTATGAGCAATTAACGATTGACCTCTGGGAAGACGCAGAAGACTTGCTCAATGAATACTT‑‑‑‑‑‑‑GGAGGAAGTCGAGGAA‑‑‑‑‑‑‑‑‑GTCG‑‑‑‑‑‑‑C‑‑ACGACAGAAAGAAATTGACCGCGCTAAGGCCCGTAAAGAACGTCACGAGGGGCGCTTAGAGGCACGCAGATTCAAACGTCGCAACCGCAAGGCACGTAAAGCACACAAAGCTAATCGCGAAAGAATGCTTGCTGCGTGGCGATGGGCTGAACGTCAAGAACGGCGTAACCATGAGGTAGCTGTAGATGTACTAGGA                                                                                                       <  1:153493/301‑1`  
                                                                                                                                                                                                                            `gcactCTGCAAGCGCGTATCTATGAGCAATTAACGATTGACGTCTGGGAAGACGCAGAAGACTTGCGAAATGAATACTT‑‑‑‑‑‑‑G‑‑‑‑‑‑‑‑‑GAGGAA‑‑‑‑‑‑‑‑‑GTCG‑‑‑‑‑‑‑C‑‑ACGACAAAAAGAAATTGACCGCGCTAAGGCCAGTAAAGACCGTCACGAGGGGCGCTTAGAGGCACGCAGATTCAAACGTCGCAACCGCAAGGCACGTAAAGCACACAAAGCTAAGCGCGAAAGAATGCTTGCTGCGTGGCGATGGGCTGAACGTCAAGAACGGCGTAACCATGAGGTAGCTGTAGATGTACTAGGAAGAACCAATAACGC                                                                                         <  1:444262/296‑1`  
                                                                                                                                                                                                                            `GCATCCTGCAAGCGCGTATCTATGAGCAATTAACGATTGACCTCTGGGAAGACGCAGAAGACTTGCTCAATGAATACTT‑‑‑‑‑‑‑G‑‑‑‑‑‑‑‑‑GAGGAA‑‑‑‑‑‑‑‑‑GTCG‑‑‑‑‑‑‑C‑‑ACGACAGAAAGAAATTGACCGCGCTAAGGCCCGTAAAGAACGTCACGAGGGGCGCTTAGAGGCACGCAGATTCAAACGTCGCAACCGCAAGGCACGTAAAGCACACAAAGCTAAGCGCGAAAGAATGCTTGCTGCGTGGCGATGGGCTGAACGTCAAGAACGGCGTAACCATGAGGTAGCTGTAGATGTACTAGGAAGAACCAATAACGC                                                                                         <  1:87498/301‑1`  
                                                                                                                                                                                                                               `TCCTGTAAGCGCGTATCTATGAGCAATTAACGATTGACCTCTGGGAAGACGCAGAAGACTTGCTCAATGAATACTT‑‑‑‑‑‑‑G‑‑‑‑‑‑‑‑‑GAGGAA‑‑‑‑‑‑‑‑‑GTCG‑‑‑‑‑‑‑C‑‑ACGACAGAAAGAAATTGACCGCGCTAAGGCCCGTAAAGAACGTCACGAGGGGCGCTTAGAGGCACGCAGATTCAAACGTCGCAACCGCAAGGCACGTAAAGCACACAAAGCTAAGCGCGAAAGAATGCTTGCTGCGTGGCGATGGGCTGAACGTCAAGAACGGCGTAACCAGGAGGTAGCTGTAGATGTACTAGGAAGAACCAATAACGCTAa                                                                                      >  1:240609/1‑300`  
                                                                                                                                                                                                                                 `CTGCAAGGGCGTATCTATGAGCAATTAACGATTGACCTCTGGGAAGACGCAGAAGACTTGCTCAATGAATACTT‑‑‑‑‑‑‑G‑‑‑‑‑‑‑‑‑GAGGAA‑‑‑‑‑‑‑‑‑GTCG‑‑‑‑‑‑‑C‑‑ACGACAGAAAGAAATTGACCGCGCTAAGGCCCGTAAAGAACGTCACGAGGGGCGCTTAGAGGCACGCAGATTCAAACGTCGCAACCGCAAGGCACGTAAAGCACACAAAGCTAAGCGCGAAAGAATGCTTGCTGCGTGGCGATGGGCTGAACGTCAAGAACGGCGTAACCATGAGGTAGCTGTAGATGTACTAGGAAGAACCAATAACGCTATGC                                                                                    <  1:407519/301‑1`  
                                                                                                                                                                                                                                   `aaagagacagATCTATGAGCAATTAACGATTGACCTCTGGGAAGACGCAGAAGACTTGCTCAATGAATACTT‑‑‑‑‑‑‑G‑‑‑‑‑‑‑‑‑GAGGAA‑‑‑‑‑‑‑‑‑GTCG‑‑‑‑‑‑‑C‑‑ACGACAGAAAGAAATTGACCGCGCTAAGGCCCGTAAAGAACGTCACGAGGGGCGCTTAGAGGCACGCAGATTCAAACGTCGCAACCGCAAGGCACGTAAAGCACACAAAGCTAAGCGCGAAAGAATGCTTGCTGCGTGGCGATGGGCTGAACGTCAAGAACGGCGTAACCATGAGGTAGCTGTAGATGTACTAGGAAGAACCAATAACGCTATGCTC                                                                                  <  1:545834/291‑1`  
                                                                                                                                                                                                                                   `GCAATCGCGGATTTAGGAGCAATTAACGATGGACATCTGGGAAGACGCAGAAGACTGGCTAAATGAATACTT‑‑‑‑‑‑‑G‑‑‑‑‑‑‑‑‑GAGGAA‑‑‑‑‑‑‑‑‑GTCG‑‑‑‑‑‑‑C‑‑ACGACAGAAAGAAATTGACCGCGCTAAGGCCCGTAAAGAACGTCACGAGGGGCGCTGAGAGGCACGCAGATTCAAACGTCGCAACCGCAAGGCACGTAAAGCACACAAAGCTAAGCGCGAAAGAATGATTGCTGCGTGGCGATGGGCTGAACGTCAAGAACGGCGTAACCATGAGGTAGCTGTAGATGTACTAGGAAGAACCAATAACGCTATGCTC                                                                                  <  1:311156/301‑1`  
                                                                                                                                                                                                                                    `aaaatgtgtataagagacagataAACGATTGACCTCTGGGAAGACGCAGAAGACTTGCTCAATGAATACTT‑‑‑‑‑‑‑G‑‑‑‑‑‑‑‑‑GAGGAA‑‑‑‑‑‑‑‑‑GTCG‑‑‑‑‑‑‑C‑‑ACGAAAGAAAGAAATTGACCGCGCTAAGGCCCGTAAAGAACGTCAGGAGGGGCGCTTAGAGGCACGAAGATTCAAACGTCGCAACCGCAAGGCACGTAAAGCACACAAAGCTAAGCGAAAAAGAATGCTTGCTGCGTGGCGATGGGCTGAACGTCAAGAACGGCGTAACCATGAGGTAGCTGTAGATGTACTAGGAAGAACCAATAACGCTATGCTCT                                                                                 <  1:204990/278‑1`  
                                                                                                                                                                                                                                       `GCGCGTATCTATGAGCAATTAACGATTGACCTCTGGGAAGACGCAGAAGACTTGCTAAATGAATACTT‑‑‑‑‑‑‑G‑‑‑‑‑‑‑‑‑GAGGAA‑‑‑‑‑‑‑‑‑GTCG‑‑‑‑‑‑‑C‑‑ACGACAGAAAGAAATTGACCGCGCTAAGGCCCGTAAAGAACGTCACGAGGGGCGCTTGGAGGCACGCAGATTCAAACGTCGCAACCGCAAGGAACGTAAAGCACACAAAGCTAAGCGCGAAAGAATGCTTGCTGCGTGGCGATGGGCTGAACGTCAAGAACGGCGTAACCATGAGGTAGCTGTAGATGTACTAGGAAGAGACAATAACGCTCTGCTCTGGG                                                                              >  1:119270/1‑301`  
                                                                                                                                                                                                                                            `aATCTATGAGCAATTAACGATTGACCTCTGGGAAGACGCAGAAGACTTGCTCAATGAATACGT‑‑‑‑‑‑‑G‑‑‑‑‑‑‑‑‑GAGGAA‑‑‑‑‑‑‑‑‑GTCG‑‑‑‑‑‑‑C‑‑ACGACAGAAAGAAATTGACCGCGCTAAGGCCCGTAAAGAACGTCACGAGGGGCGCTTAGAGGCACGCAGATTCAAACGTCGCAACCGCAAGGCACGTAAAGCACACAAAGCTAAGCGCGAAAGAATGCTTGCTGCGTGGCGATGGGCTGAACGTCAAGAACGGCGTAACCATGAGGTAGCTGTAGATGTACTAGGAAGAACCAATAACGCTATGCTCTGGGTaaca                                                                         <  1:133307/300‑5`  
                                                                                                                                                                                                                                            `TATCAATGAGCAATTAACGATTGACCTCTGGGAAGACGAAGAAGACTAGCTCAATGAATACTT‑‑‑‑‑‑‑G‑‑‑‑‑‑‑‑‑GAGGAA‑‑‑‑‑‑‑‑‑GTCGAGGAAGTCGAACGACAGAAAGAAATTGACCGCGCTAAGGCCCGTAAAGAACGTCACGAGGGGCGCTTAGAGGCACGCAGATTCAAACGTCGCAACCGCAAGGCACGTAAAGCACACAAAGCTAAGCGCGAAAGAATGCTTGCTGCGTGGCGATGGGCTGAACGTCAAGAACGGCGTAACCATGGGGTAGCTGTAGATGTACTAGGAAGAACCAATAACGCTATGCTC                                                                                  <  1:617459/301‑1`  
                                                                                                                                                                                                                                             `ATCTATGAGCAATTAACGATTGACCTCTGGGAAGACGCAGAAGACTTGCTCAATGAATACTT‑‑‑‑‑‑‑GGAGGAAGTCGAGGAA‑‑‑‑‑‑‑‑‑GTCG‑‑‑‑‑‑‑C‑‑ACGACAGAAAGAAATTGACCGCGCTAAGGCCCGTAAAGAACGTCACGAGGGGCGCTTAGAGGCACGCAGATTCAAACGTCGCAACCGCAAGGCACGTAAAGCACACAAAGCTAAGCGCGAAAGAATGCTTGCTGCGTGGCGATGGGCTGAACGTCAAGAACGGCGTAACCATGAGGTAGCTGTAGATGTACTAGGAAGAACCAATAACGCTATGCTCT                                                                                 >  1:604091/1‑301`  
                                                                                                                                                                                                                                             `ATCTATGAGCAATTAACGATTGACCTCTGGGAAGACGCAGAAGACTTGCTCAATGAATACTT‑‑‑‑‑‑‑G‑‑‑‑‑‑‑‑‑GAGGAA‑‑‑‑‑‑‑‑‑GTCG‑‑‑‑‑‑‑C‑‑ACGACAGAAAGAAATTGACCGCGCTAAGGCCCGTAAAGAACGTCACGAGGGGCGCTTAGAGGCACGCAGATTCAAACGTCGCAACCGCAAGGCACGTAAAGCACACAAAGCTAAGCGCGAAAGAATGCTTGCTGCGTGGCGATGGGCTGAACGTCAAGAACGGCGTAACCATGAGGTAGCTGTAGATGTACTAGGAAGAACCtgtctcttatacacatctccgagcc                                                                        >  1:373284/1‑276`  
                                                                                                                                                                                                                                               `CTATGAGCAATTAACGATTGACCTCTGGGAAGACGCAGAAGACTTGCTCAATGAATACTT‑‑‑‑‑‑‑GGAGGAAGTCGAGGAA‑‑‑‑‑‑‑‑‑GTCG‑‑‑‑‑‑‑C‑‑ACGACAGAAAGCAATTGACCGCGCTAAGGCCCGTAGAGAACGTCACGAGGGGCGATTAGAGGCACGCAGATTCAAACGTCGCAACCGCAAGGCACGTAAAGCACACCAAGCTAAGCGCGAAAGAATGCTTGCTGCGTGGCGATGGGCTGAACGTCAAGAACGGCGTAACCATGAGGTAGCTGTAGATGTACTAGGAAGAACCAAGACCGCTATGCTCTGG                                                                               >  1:215432/1‑301`  
                                                                                                                                                                                                                                                    `AGCAATTAACGATTGACCTCTGGGAAGACGCAGAAGACTTGCTCAATGAATACTT‑‑‑‑‑‑‑G‑‑‑‑‑‑‑‑‑GAGGAA‑‑‑‑‑‑‑‑‑GTCG‑‑‑‑‑‑‑C‑‑ACGACAGAAAGAAATTGACCGCGCTAAGGCCCGTAAAGAACGTCACGAGGGGCGCTTAGAGGCACGCAGATTCAAACGTCGCAACCGCAAGGCACGTAAAGCACACAAAGCTAAGCGCGAAAGAATGCTTGCTGCGTGGCGATGGGCTGAACGTCAAGAACGGCGTAACCATGAGGTAGCTGTAGATGTACTAGGAAGAACCAATAACGCTATGCTCTGGGTCAACATGTTCTC                                                                 <  1:611609/301‑1`  
                                                                                                                                                                                                                                                      `CAATTAACGATTGACCTCTGGGAAGACGCAGAAGACTTGCTCAATGAATACTT‑‑‑‑‑‑‑G‑‑‑‑‑‑‑‑‑GAGGAA‑‑‑‑‑‑‑‑‑GTCG‑‑‑‑‑‑‑C‑‑ACGACAGAAAGAAATTGACCGCGCTAAGGCCCGTAAAGAACGTCACGAGGGGCGCTTAGAGGCACGCAGATTCAAACGTCGCAACCGCAAGGCACGTAAAGCACACAAAGCTAAGCGCGAAAGAATGCTTGCTGCATGGCGATGGGCTGAACGTCAAGAACGGCGTAACCATGAGGTAGCTGTAGATGTACTAGGAAGAACCAATAACGCTATGCTCTGGGTCAACATGTTCTCct                                                               >  1:469476/1‑299`  
                                                                                                                                                                                                                                                        `ATTAACGATTGACCTCTGGGAAGACGCAGAAGACTTGCTCAATGAATACTT‑‑‑‑‑‑‑G‑‑‑‑‑‑‑‑‑GAGGAA‑‑‑‑‑‑‑‑‑GTCG‑‑‑‑‑‑‑C‑‑ACGACAGAAAGAAATTGACCGCGCTAAGGCCCGTAAAGAACGTCACGAGGGGCGCTTAGAGGCACGCAGATTCAAACGTCGCAACCGCAAGGCACGTAAAGCACACAAAGCTAAGCGCGAAAGAATGCTTGCTGCGTGGCGATGGGCTGAACGTCAAGAACGGCGTAACCATGAGGTAGCTGTAGATGTACTAGGAAGAACCAATAACGCTATGCTCTGGGTCAACATGTTCTCTGGG                                                             >  1:506099/1‑301`  
                                                                                                                                                                                                                                                             `CGATTGCCCTCGGGGAAGACGCAGAAGACTTGCTCAATGAATACTT‑‑‑‑‑‑‑GGAGGAAGTCGAGGAA‑‑‑‑‑‑‑‑‑GTCG‑‑‑‑‑‑‑C‑‑ACGAAAGAAAGAAATTGACCGCGCTAAGGCCCGTAAAGAACGTCACGAGGGGCGCTTAGAGGCACGCAGATTCAAACGTCGCAACCGCAAGGCACGTAAAGCACACAAAGCTAAGCGCGAAAGAATGCTTGCTGCGTGGCGATGGGCTGAACGTCAAGAACGGCGTAACCATGAGGTAGCTGTAGATGTACTAGGAAGAACCAATAACGCTATGATCTGGGTCAACATGTTCTC                                                                 <  1:165113/301‑1`  
                                                                                                                                                                                                                                                             `CGATTGACCTCTGGGAAGACGCAGAAGACTTGCTCAATGAATACTT‑‑‑‑‑‑‑G‑‑‑‑‑‑‑‑‑GAGGAA‑‑‑‑‑‑‑‑‑GTCG‑‑‑‑‑‑‑C‑‑CCGACAGAAAGATATTGACCGCGCTAAGGCCCGTAAAGAACGTCACGAGGGGCGCTTAGAGGCACGCAGATTCAAACGTCGCAACCGCAAGGCCCGTAAAGCACACAAAGCTAAGCGCGAAAGAATGCTTGCTGCGTGGCGATGGGCTGCACGTCAAGAACGGCGTAACCATGAGGTAGCTGTAGATGTACTAGGAAGAACCAATAACGCTATGCTCTGGGTCAACATGTTCTCTGGGTACTT                                                        >  1:639067/1‑301`  
                                                                                                                                                                                                                                                                         `GGGAAGACGCAGAAGACTTGCTCAATGAATACTT‑‑‑‑‑‑‑G‑‑‑‑‑‑‑‑‑GAGGAA‑‑‑‑‑‑‑‑‑GTCG‑‑‑‑‑‑‑C‑‑ACGACAGAAAGAAATTGACCGCGCTAAGGCCCGTAAAGAACGTCACGAGTGGCGCTTAGAGGTACGCAGATTCAAACGTCGCAACCGCAAGGCACGTAAAGCACACAAAGCTAAGCGCGCAAGAATGCTTGCTGCGTGGCGATGGGCTGAACGTCAAGAACGGCGTAACCATGAGGTAGCTGTAGATGTACTAGGAAGAACCAATAACGCTATGCTCTGGGTCAACATGTTCGCTGGGGACTTTAAGGCGCTTGA                                            >  1:125806/1‑301`  
                                                                                                                                                                                                                                                                           `GAAGACGCAGAAGACTTGCTCAATGAAGACTT‑‑‑‑‑‑‑GGAGGAAGTCGAGGAA‑‑‑‑‑‑‑‑‑GTCG‑‑‑‑‑‑‑C‑‑ACTACAGAAAGAAATGGACCGCGCTAATGCCCGTAAAGAACGTCACGAGGGGCGCTTAGAGGCACGCAGATGCAAACGTCGCAACCGCAAGGCACGTAAGGCACACAAAGCTAAGCGCGAAAGAATGATTGCTGCGTGGCGATGGGCTGAACGTCAAGAACGGCGTAACCATGAGATAGCTGTAGATGTACTAGGAAGAACCAATAACGCTATGCTCTGGGTCAACATGTTCTCTGGGGACTTTAAGG                                                   <  1:500029/301‑1`  
                                                                                                                                                                                                                                                                             `gagatgtgtataagaggcagATGAAGACTT‑‑‑‑‑‑‑G‑‑‑‑‑‑‑‑‑GAGGAA‑‑‑‑‑‑‑‑‑GTCG‑‑‑‑‑‑‑C‑‑ACGACAGAAAGAAATTGACCGCGATAAGGCCCGTAAAGAACGTCACGAGGGGCGCTTAGAGGCACGCAGATTCAAACGTCGCAACCGCAAGGCACGTAAAGCACACAAAGCTAAGCGCGAAAGAATGCTAGCTGCGTGGCGATGGGCTGAACGTCAAGAACTGCGTAACCATGAGGTAGCTGTAGATGTACTAGGAAGAACCAATAACGCTATGCTCTGGGTCAACATGTTCTCTGGGGACTTTAAGGCGCTTGAGGAA                                        <  1:246936/281‑1`  
                                                                                                                                                                                                                                                                                `CGCAGAAGACTTGCTCAATGAATACCT‑‑‑‑‑‑‑G‑‑‑‑‑‑‑‑‑GAGGAA‑‑‑‑‑‑‑‑‑GTCG‑‑‑‑‑‑‑C‑‑AAGACAGAAAGAAATTGACCGCGCTAAGGCCCGTAAAGAACGTCACGAGGGGCGCTTAGAGGCACGCAGATTCAAACGTCGCAACCGCAAGGCACGTAAAGAACACAAAGCTAAGCGCGAAAGAATGCCTGCTGCGTGGCGATGGGCTGAACGTCAAGAACGGCGTAACCATGAGGTAGCTGTAGATGTACTAGGAAGAACCAATAACGATATGCTCTGGGTCAACATGTTCTCTGGGGACTTTAAGGAGCTTGAGGAACGA                                     >  1:34213/1‑301`  
                                                                                                                                                                                                                                                                                    `GAAGACTTGCTCAATGAATACTT‑‑‑‑‑‑‑G‑‑‑‑‑‑‑‑‑GAGGAA‑‑‑‑‑‑‑‑‑GTCG‑‑‑‑‑‑‑C‑‑ACGACAGAAAGAAATTGACCGCGCTAAGGCCCGTAAAGAACGTCACGAGGGGCGCTTAGAGGCACGCAGATTCAAACGTCGCAACCGCAAGGCACGTAAAGCACACAAAGCTAAGCGCGAAAGAATGCTTGCTGCGTGGCGATGGGCTGAACGTCAAGAACGGCGTAACCATGAGGTAGCTGTAGATGTACTAGGAAGAACCAATAACGCTATTCTCTGGGTCCACATGTTCTCTGGGGACTTTAAGGCCCTTGAGGAACGAATCc                                 >  1:305338/1‑300`  
                                                                                                                                                                                                                                                                                            `GCTCAATGAATACTT‑‑‑‑‑‑‑G‑‑‑‑‑‑‑‑‑GAGGAA‑‑‑‑‑‑‑‑‑GTCG‑‑‑‑‑‑‑C‑‑ACGACAGAAAGAAATTGACCGCGCTAAGGCCCGTAAAGAACGTCACGAGGGGCGCTTAGAGGCACGCAGATTCAAACGTCGCAACCGCAAGGCACGTAAAGCACACAAAGCTAAGCGCGAAAGAATGCTTGCTGCGTGGCGATGGGCTGAACGTCAAGAACGGCGTAACCATGAGGTAGCTGTAGATGTACTAGGAAGAACCAATAACGCTATGCTCTGGGTCAACATGTTCTCTGGGGACTTTAAGGCGCTTGAGGAACGAATCGCGCTGCAC                         >  1:584808/1‑301`  
                                                                                                                                                                                                                                                                                             `CTCAATGAATACGT‑‑‑‑‑‑‑G‑‑‑‑‑‑‑‑‑GAGGAA‑‑‑‑‑‑‑‑‑GTCG‑‑‑‑‑‑‑C‑‑ACGACAGAAAGAAATTGACCGCGCTAAGGCCCGTAAAGAACGTCACGAGGGGCGCTTAGAGGCACGCAGATTCAAACGTCGCAACCGCAAGGCACGTAAAGCACACAAAGCTAAGCGCGAAAGAATGCTTGCTGCGTGGCGATGGGCTGAACGTCAAGAACGGCGTAACCATGAGGTAGCTGTAGATGTACTAGGAAGAACCAATAACGCTATGCTCTGGGTCAACATGTTCTCTGGGGACTTTAAGGCGCTTGAGGAACGAATCGCGCTGCACT                        <  1:322396/301‑1`  
                                                                                                                                                                                                                                                                                               `acagGAATACTT‑‑‑‑‑‑‑G‑‑‑‑‑‑‑‑‑GAGGAA‑‑‑‑‑‑‑‑‑GTCG‑‑‑‑‑‑‑C‑‑ACGACAGAAAGAAATTGACCGCGCTAAGGCCCGTAAAGAACGTCACGAGGGGCGCTTAGAGGCACGCAGATTCAAACGTCGCAACCGCAAGGCACGTAAAGCACACAAAGCTAAGCGCGAAAGAATGCTTGCTGCGTGGCGATGGGCTGAACGTCAAGAACGGCGTAACCATGAGGTAGCTGTAGATGTACTAGGAAGAACCAATAACGCTATGCTCTGGGTCAACATGTTCTCTGGGGACTTTAAGGCGCTTGAGGAACGAATCGCGCTGCACTGG                      <  1:399900/297‑1`  
                                                                                                                                                                                                                                                                                                       `aaTT‑‑‑‑‑‑‑G‑‑‑‑‑‑‑‑‑GAGGAA‑‑‑‑‑‑‑‑‑GTCG‑‑‑‑‑‑‑C‑‑ACGACAGAAAGAAATTGCACGCGTTAAGGCCCGAAAAGAACGTGACGAGGGGCGGTTAGAGGCACGCAGATTCAAACGTCGCAACCGCAAGGCACGTAAAGCACACAAAGCTAAGCGAGAAAGAATGCTTGCTGCGTGGCGATGGGAGTAACGTGAAGAACGGCGTAACCATTAGGTAGCTGTAGATGTACTATGAAGAACCAATAACGCTATGCTCTGGGTCAACAGGGTCGCTGGGGACTTTAAGGCGCTTGAGGAACGAATCGCGCTGCACTGGCGTAATGC              <  1:235625/299‑1`  
                                                                                                                                                                                                                                                                                                       `ACTT‑‑‑‑‑‑‑G‑‑‑‑‑‑‑‑‑GAGGAA‑‑‑‑‑‑‑‑‑GTCG‑‑‑‑‑‑‑C‑‑ACGACAGAAAGAAATTGACCGCGCTAAGGCCCGTAAAGAACGTCACGAGGGGCGCTTAGAGGCACGCAGATTCAAACGTCGCAACCGCAAGGCACGTAAAGCACACAAAGCTAAGCGCGAAAGAATGCTTGCTGCGTGGCGATGGGCTGAACGTCAAGAACGGCGTAACCATGAGGTAGCTGTAGATGTACTAGGAAGAACCAATAACGCTATGCTCTGGGTCAACCTGTTCCCTGGGGACTTTAAGGCGCTTGAGGAACGAATCGCCCTTCACTTGCGTAATGt              >  1:628935/1‑300`  

                                                                                                                                                                                                                                                                                                                                                                                                                                                                                                                                                                                                                                                                    
`ACTTACAACAACGTTTTCGACCACGCTTACGAAATGCTGAAAGAAAACATCCGTTATGATGACATCCGTGACACTGATGACCTGCACGATGCTATTCACATGGCTGCCGATAATGCAGTTCCGCACTACTACGCTGACATCTTTAGCGTAATGGCAAGTGAGGGCATTGACCTTGAGTTCGAAGACTCTGGTCTGATGCCTGACACCAAGGACGTAATCCGCATCCTGCAAGCGCGTATCTATGAGCAATTAACGATTGACCTCTGGGAAGACGCAGAAGACTTGCTCAATGAATACTT‑‑‑‑‑‑‑G‑‑‑‑‑‑‑‑‑GAGGAA‑‑‑‑‑‑‑‑‑GTCG‑‑‑‑‑‑‑‑‑‑‑‑‑‑‑‑‑‑‑‑‑‑‑‑‑‑‑‑‑‑‑‑‑‑‑‑‑‑‑‑‑‑‑‑‑‑‑‑‑‑‑‑‑‑‑‑‑‑‑‑‑‑‑‑‑‑‑‑‑‑‑‑‑‑‑‑‑‑‑‑‑‑‑‑‑‑‑‑‑‑‑‑‑‑‑‑‑‑‑‑‑‑‑‑‑‑‑‑‑‑‑‑‑‑‑‑‑‑‑‑‑‑‑‑‑‑‑‑‑‑‑‑‑‑‑‑‑‑‑‑‑‑‑‑‑‑‑‑‑‑‑‑‑‑‑‑‑‑‑‑‑‑‑‑‑‑‑‑‑‑‑‑‑‑‑‑‑‑‑‑‑‑‑‑‑‑‑‑‑‑‑‑‑‑‑‑‑‑‑‑‑‑‑‑‑‑‑‑‑‑‑‑‑‑‑‑‑‑‑‑‑‑‑‑‑‑‑‑‑‑‑‑‑‑‑‑‑‑‑‑‑‑‑‑‑‑‑‑‑‑‑‑‑‑‑‑‑‑‑‑‑‑‑‑‑‑‑‑‑‑‑‑‑‑‑‑‑‑‑‑‑‑‑‑‑‑‑‑‑‑‑‑‑‑‑‑‑‑‑‑‑‑‑‑‑‑‑  >  NC_001604.1/943‑1252`  
`‑‑‑‑‑‑‑‑‑‑‑‑‑‑‑‑‑‑‑‑‑‑‑‑‑‑‑‑‑‑‑‑‑‑‑‑‑‑‑‑‑‑‑‑‑‑‑‑‑‑‑‑‑‑‑‑‑‑‑‑‑‑‑‑‑‑‑‑‑‑‑‑‑‑‑‑‑‑‑‑‑‑‑‑‑‑‑‑‑‑‑‑‑‑‑‑‑‑‑‑‑‑‑‑‑‑‑‑‑‑‑‑‑‑‑‑‑‑‑‑‑‑‑‑‑‑‑‑‑‑‑‑‑‑‑‑‑‑‑‑‑‑‑‑‑‑‑‑‑‑‑‑‑‑‑‑‑‑‑‑‑‑‑‑‑‑‑‑‑‑‑‑‑‑‑‑‑‑‑‑‑‑‑‑‑‑‑‑‑‑‑‑‑‑‑‑‑‑‑‑‑‑‑‑‑‑‑‑‑‑‑‑‑‑‑‑‑‑‑‑‑‑‑‑‑‑‑‑‑‑‑‑‑‑‑‑‑‑‑‑‑‑‑‑‑‑‑‑‑‑‑‑‑‑‑‑‑‑‑‑‑‑‑‑‑‑‑‑‑‑‑‑‑‑‑‑‑‑‑‑‑‑‑‑‑‑‑‑‑‑‑‑‑‑‑‑‑‑‑‑‑‑‑‑‑‑‑‑‑‑‑‑‑‑‑‑gaggaa‑‑‑‑‑‑‑‑‑gtcg‑‑‑‑‑‑‑C‑‑ACGACAGAAAGAAATTGACCGCGCTAAGGCCCGTAAAGAACGTCACGAGGGGCGCTTAGAGGCACGCAGATTCAAACGTCGCAACCGCAAGGCACGTAAAGCACACAAAGCTAAGCGCGAAAGAATGCTTGCTGCGTGGCGATGGGCTGAACGTCAAGAACGGCGTAACCATGAGGTAGCTGTAGATGTACTAGGAAGAACCAATAACGCTATGCTCTGGGTCAACATGTTCTCTGGGGACTTTAAGGCGCTTGAGGAACGAATCGCGCTGCACTGGCGTAATGCTGACCGGATGGC  >  NC_001604.1/2742‑3039`

  

Alignment Legend

Aligned base mismatch/match (shaded by quality score): `ATCG/ATCG` `< 3 ≤` `ATCG/ATCG` `< 9 ≤` `ATCG/ATCG` `< 20 ≤` `ATCG/ATCG` `< 34 ≤` `ATCG/ATCG` `< 38 ≤` `ATCG/ATCG`

Unaligned base: `atcg`    Masked matching base: `atcg`    Alignment gap: `‑`    Deleted base: `‑`

Reads not counted as support for junction

read\_name Not counted due to insufficient overlap past the breakpoint.

read\_name Not counted due to not crossing MOB target site duplication.
